# An Unusual Case of Extensive Cavernous Hemangioma of the Nasopharynx

**DOI:** 10.22038/ijorl.2019.35143.2159

**Published:** 2020-01

**Authors:** Pradeep Pradhan, Swagatika Samal, Chappity Preetam

**Affiliations:** 1 *Department of Otorhinolaryngology, All India Institute of Medical Sciences, Bhubaneswar, Odisha, India*, *Pin-751019.*; 2 *Department of Pathology and laboratory medicine, All India Institute of Medical Sciences, Bhubaneswar, Odisha, India*

**Keywords:** Cavernous hemangioma, Endoscopic excision, Nasopharynx

## Abstract

**Introduction:**

Giant cavernous hemangioma involving the nose extending to the nasopharynx and oropharynx with complete obstruction of the airway is very unusual and is yet to be described in the literature. In the present case, we have described a giant cavernous hemangioma successfully managed with endoscopic excision.

**Case Report:**

A 38-year-old male patient presented with recurrent nasal bleeding for 24 months and progressive obstruction of the right nasal cavity for 8 months. Diagnostic nasal endoscopy showed a greyish mass filling the whole of the right nasal cavity and contrast-enhanced CT scan of the nose and paranasal sinus revealed a large heterogeneous contrast enhanced lesion in the nasal cavity. The endoscopic biopsy was suggestive of cavernous hemangioma. Endoscopic excision was done and the patient has been followed up for the past 12 months without any recurrence of the disease.

**Conclusion:**

Cavernous hemangioma is an uncommon benign entity of the nose and paranasal sinus. Due to the nonspecific clinical and radiological pictures, it is often a challenge for the preoperative diagnosis. A high index of suspicion and complete understanding of the clinicopathological profile of the patient is vital as the major differential diagnosis is the sinonasal malignancy simulating with a similar clinical picture.

## Introduction

Although hemangiomas are common vascular malformation in the head and neck, sinonasal hemangiomas are rarely encountered in common clinical practice (1). The majority of the lesions are of capillary type, predominantly found in the paediatric population and the common subsites are the septum and the vestibule (2). In contrast, cavernous hemangiomas are very rare as described in the current literature, mostly affecting adult females and the common site is the lateral wall of the nose (3-7). Although isolated cavernous hemangioma involving the maxillary sinus and nasal cavity have been described in the past, a giant cavernous hemangioma involving the nose extending to the nasopharynx and oropharynx with complete obstruction of the airway is very unusual and is yet to be described in the literature. It is always a challenge to diagnose the disease clinically and radiologically because of the nonspecific clinical, radiological features. Here we have presented a rare case of a giant sinonasal cavernous hemangioma in an adult male presented with recurrent epistaxis which was successfully managed by endoscopic excision.

## Case Report

**Fig 1:**
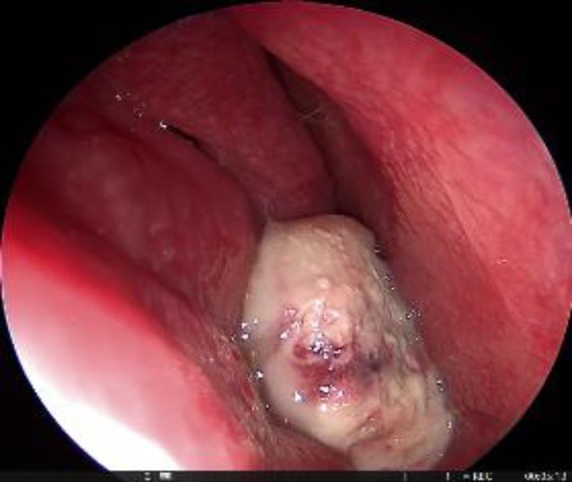
Diagnostic nasal endoscopy showed a greyish mass filling the whole of the right nasal

A 38-year-old male patient presented with recurrent nasal bleeding for 24 months and progressive obstruction of the right nasal cavity for 8 months. Anterior rhinoscopy did not reveal any significant abnormality except an organized clot and diagnostic nasal endoscopy showed a greyish mass filling the whole of the right nasal cavity extending posteriorly towards the nasopharynx (Fig.1).

**Fig 2:**
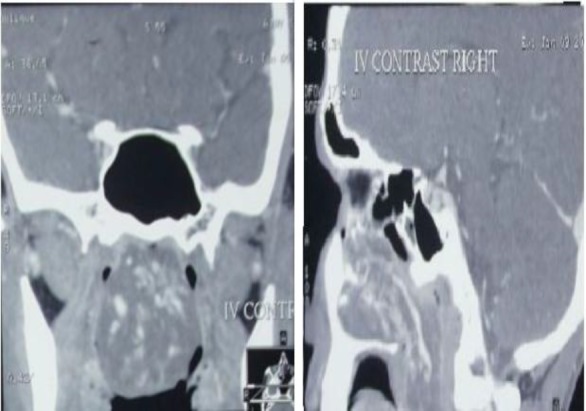
Shows contrast enhanced CT scan (Coronal and Sagittal cut) demonstrating a heterogeneous enhanced mass occluding the whole nasopharynx hanging to the oropharynx

Contrast-enhanced CT scan of the nose and paranasal sinus revealed a large heterogeneous contrast enhanced lesion filling the whole of the right nasal cavity extending the nasopharynx hanging into the oropharynx (Fig.2). 

**Fig 3:**
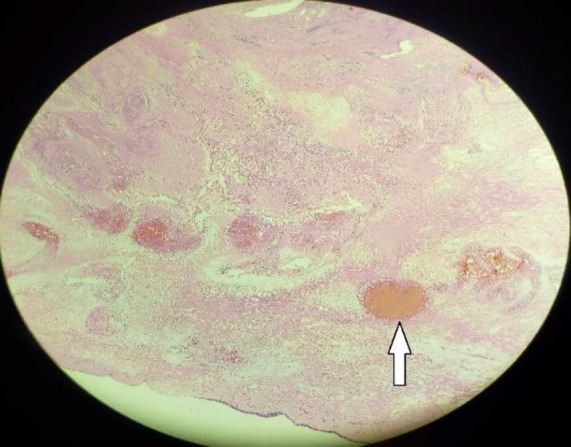
Microscopic section shows the respiratory epithelium. White arrow shows the sub-epithelial tissue, consisting of multiple dilated vascular channels of varying caliber, containing blood and fibrin, lined by flattened endothelium (H& E 20X)

Routine hematological examinations, including the coagulation profile, were found to be normal. Keeping in mind the possibility of sinonasal malignancy, the endoscopic biopsy was taken which revealed multiple dilated vascular channels of varying caliber, containing blood and fibrin, lined by flattened endothelium in the sub-epithelial tissue (H& E 20X),confirmed to be a cavernous hemangioma (Fig.3). 

**Fig 4:**
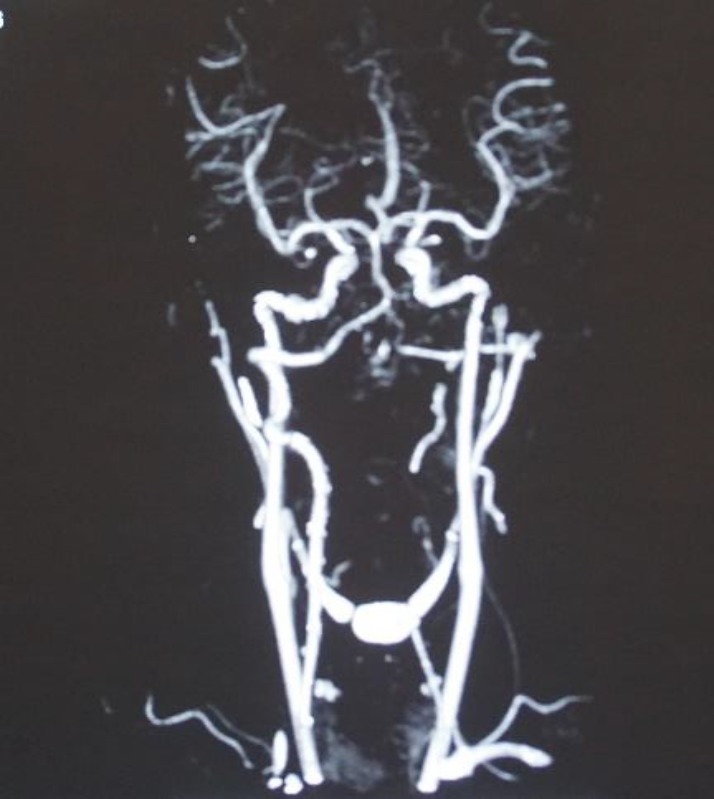
CT angiography revealed the absence of definitive feeding vessel supplying the tumor

The histological feature was suggestive of cavernous hemangioma. CT angiography was advised which did not reveal any dominant feeding vessel of the tumor (Fig. 4).

After the informed written consent, the patient was planned for the endoscopic excision of the tumor under general anaesthesia. The tumor was completely excised through endoscopic modified Denker’s approach without any significant blood loss. There were no significant intraoperative or postoperative complications detected in the patient. The final histopathology was confirmed to be the same as that of the preoperative diagnosis. The patient has been in regular follow-up for the past 12 months with serial nasal endoscopy without the recurrence of symptoms.

## Discussion

Although capillary hemangioma is very often encountered in the nose and paranasal sinuses, cavernous hemangiomas are very rare and only a hand full of cases have been described in the literature (4–9). Clinical presentations depend upon the site and extension of the tumor, which includes rhinorrhea, unilateral nasal obstruction, epistaxis, and features of obstructive sleep apnea (OSA) and later is more prominent when there is complete obstruction of the nasopharynx. 

On diagnostic nasal endoscopy (DNE), it usually presents a pedunculated or sessile greyish/reddish mass in the nose with the special predilection to the females in their second to the third decade of life (4-7). 

In contrast, our patient was a 38 year male presented with complete nasal obstruction with the history of intermittent epistaxis and on DNE, the mass was found to occlude the while of the choana. In contrast enhanced CT scan, it usually appears as a non-homogenous mass due to the presence of hemorrhage and necrosis in most of the cases. Like a malignant lesion, it can cause thinning and erosion of the bone and can compress the adjacent structure (10). In the current case, although the tumor was very extensive it did not have the features of bony erosion or thinning of the bone in the CT scan. Angiography may be useful for pre-operative embolization to reduce intraoperative bleeding. In our patient, although the tumor was vascular we did not find the definitive feeding vessel and hence patient did not undergo embolization.

Treatment is primarily surgical, ranging from complete excision to local resection sparing the vital structures and the approaches could be open, endoscopic or combined depending on the extent of the lesion (11,12). Overall, the prognosis of cavernous hemangioma after the surgery is encouraging. Though data from long-term follow-up is missing, it seems evident that cavernous hemangiomas seldom recur following complete excision, and malignant transformation is unknown. A high index of suspicion is always required to diagnose the paranasal hemangioma because of atypical clinical and radiological features. Again chance and bleeding are often unpredictable and the requirement preoperative embolization is not very clear. A complete understanding of the clinicopathological profile of the patient is vital as the major differential diagnosis is the sinonasal malignancy simulating with a similar clinical picture.

## Conclusion

Cavernous hemangioma is an uncommon benign entity of the nose and paranasal sinus. Due to the nonspecific clinical and radiological pictures, it is often a challenge for preoperative diagnosis. 

A high index of suspicion and a complete understanding of the clinicopathological profile of the patient is vital as the major differential diagnosis is the sinonasal malignancy simulating with a similar clinical picture.
